# Evaluating the Quality of Health Information: Comparison of Human and Artificial Intelligence

**DOI:** 10.1111/nmo.70164

**Published:** 2025-09-24

**Authors:** Dhruva Arcot, Neha Pondicherry, Subhankar Chakraborty

**Affiliations:** ^1^ Grizzell Middle School Dublin Ohio USA; ^2^ The Ohio State University Columbus Ohio USA; ^3^ Division of Gastroenterology, Hepatology and Nutrition The Ohio State University Columbus Ohio USA

**Keywords:** artificial intelligence, ChatGPT, Copilot, DISCERN, health information, irritable bowel syndrome, social media, TikTok

## Abstract

**Background:**

Over half of all Americans seek health‐related information online, yet the quality of this digital content remains largely unregulated and variable. The DISCERN score, a validated 15‐item instrument, offers a structured method to assess the reliability of written health information. While expert‐assigned DISCERN scores have been widely applied across various disease states, whether artificial intelligence (AI) can automate this evaluation remains unknown. Specifically, it is unclear whether AI‐generated DISCERN scores align with those assigned by human experts. Our study seeks to investigate this gap in knowledge by examining the correlation between AI‐generated and human‐assigned DISCERN scores for TikTok videos on Irritable Bowel Syndrome (IBS).

**Methods:**

A set of 100 TikTok videos on IBS previously scored using DISCERN by two physicians was chosen. Sixty‐nine videos contained transcribable spoken audio, which was processed using a free online transcription tool. The remaining videos either featured songs or music that were not suitable for transcription or were deleted or were not publicly available. The audio transcripts were prefixed with an identical prompt and submitted to two common AI models—ChatGPT 4.0 and Microsoft Copilot for—DISCERN score evaluation. The average DISCERN score for each transcript was compared between the AI models and with the mean of the DISCERN score given by the human reviewers using Pearson correlation (*r*) and Kruskal Wallis test.

**Results:**

There was a significant correlation between human and AI‐generated DISCERN scores (*r* = 0.60–0.65). When categorized by the background of the content creators—medical (*N* = 26) versus non‐medical (*N* = 43), the correlation was significant only for content made by non‐medical content creators (*r* = 0.69–0.75, *p* < 0.001). Correlation between ChatGPT and Copilot DISCERN scores was stronger for videos by non‐medical content creators (*r* = 0.66) than those by medical content creators (*r* = 0.43). On linear regression, ChatGPT's DISCERN scores explained 55.6% of the variation in human DISCERN scores for videos by non‐medical creators, compared to 8.9% for videos by medical creators. For Copilot, the corresponding values were 47.2% and 9.3%.

**Conclusion:**

AI models demonstrated moderate alignment with human‐assigned DISCERN scores for IBS‐related TikTok videos, but only when content was produced by non‐medical creators. The weaker correlation for content produced by those with a medical background suggests limitations in current AI models' ability to interpret nuanced or technical health information. These findings highlight the need for further validation across broader topics, languages, platforms, and reviewer pools. If refined, AI‐generated DISCERN scoring could serve as a scalable tool to help users assess the reliability of health information on social media and curb misinformation.

## Graphical abstract

AI (ChatGPT, Copilot) DISCERN scores align closely with human DISCERN scores for TikTok videos on irritable bowel syndrome created by non‐medical creators but not for videos created by people with medical backgrounds. This highlights AI's potential in assessing health information quality, with further validation needed across diverse topics and platforms.

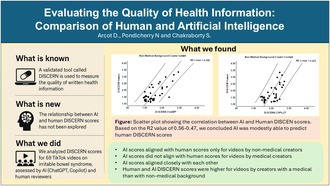

**Summary**

- Many people turn to social media for health information, but it's hard to know if that information is trustworthy.
- Our study found that artificial intelligence (AI) tools can help evaluate the quality of health content—especially when it's made by non‐medical creators.
- Using AI to rate online health videos could help people make better‐informed decisions and avoid misinformation.

## 1 Introduction

According to the Center for Disease Control and Prevention, roughly 58.5% or more than half of all Americans use the internet to look up health information [1]. Social media has become an integral part of modern life today. One study estimated that the number of new social media users grew by 150 million between 2022 and 2023—a 3.2% increase over the previous year. This amounts to approximately 410,000 new social media users every day, or nearly 5 new users joining every second [2].

The most popular social media platforms include Meta (formerly Facebook), Instagram, YouTube, TikTok, LinkedIn, Snapchat, and X (formerly Twitter). People use social media for various reasons, including seeking health‐related information. Some of these platforms are more popular than others for seeking information regarding health‐related topics. According to Harvard Public Health, about 1 in 3 Americans consult YouTube, while 1 in 5 turns to TikTok to diagnose the cause of their symptoms prior to seeing a doctor [3].

While social media has revolutionized the way information is shared, the ease of content creation and lack of quality checks means that it is also prone to misinformation [4]. A recent article described how large language models can create blog posts with disinformation in a short span of time, with scientific‐appearing references and realistic sounding patient and physician testimonials [5]. Large language models like ChatGPT, Gemini Pro, and Llama 2 were demonstrated to generate health disinformation blogs when prompted to generate content claiming that sunscreens caused skin cancer, highlighting that safeguards and oversight are important to prevent mass generation of health disinformation [6]. However, a recent review article suggests that AI's capacity to verify information is improving and, in collaboration with human oversight, there is a potential to generate AI solutions that can handle misinformation more effectively [7]. There are several tools that can be applied to measure the quality of digital health information, such as that on social media. These include the DISCERN instrument, HONcode (Health on the Net Foundation Code of Conduct), the JAMA (Journal of the American Medical Association) criteria, PEMAT (Patient Education Materials Assessment Tool) and readability measures like the Flesch–Kincaid Readability test and the SMOG (Simple Measure of Gobbledygook) Readability Formula [8, 9, 10, 11]. The DISCERN instrument is a validated, widely used tool to help users understand the quality of written consumer health information, particularly regarding treatment choices. It is a questionnaire comprising 15 items, scored on a 5‐point scale. On this scale, a score of 1 is very poor while a score of 5 is excellent. The average DISCERN score is used to measure the overall quality of the information. This tool has been widely used by healthcare professionals, researchers, patient advocacy groups, policymakers, and health organizations to evaluate the quality of health information content and digital content [12, 13, 14].

The DISCERN instrument has been applied to measure the quality of health information on social media in several diseases. A study on spine surgery information videos on TikTok found that the DISCERN score was higher for content created by health professionals [15]. Another showed that Instagram videos regarding psoriasis treatment were mostly of poor quality, despite 1 in 3 being produced by healthcare professionals [13]. A third study found that social media videos on mesh use in inguinal hernia repair were mostly of low quality [16].

Further, research has also shown that there is an inverse correlation between DISCERN scores and the popularity of a video. For example, the most popular videos about knee instability on TikTok usually had a low DISCERN score [17]. Similarly, there was an inverse correlation between quality measured by the DISCERN score and the popularity of social media videos on glaucoma [18] and microtia [19]. These studies involved experts reviewing each video and manually calculating their DISCERN scores. However, having an expert review of every piece of health information content on a given topic is neither practical nor feasible.

Artificial intelligence (or AI) is defined as a technology that enables computers and machines to simulate human learning, comprehension, problem solving, decision making, creativity, and autonomy [20]. Generative AI is a form of AI that can create original content, such as text, images, video, audio, or software code in response to a user's prompt or request [21]. ChatGPT, Copilot, and Gemini are some of the free and popular generative AI tools.

Despite the growing reliance on AI in healthcare, research into AI‐driven assessment of the quality of health‐related information is limited. A recent study assessed the accuracy of ChatGPT in evaluating health news quality, revealing that while its explanations were clear and contextually relevant, its rating accuracy varied significantly across different criteria [22]. One area of growing research has been the development of several instruments designed to evaluate the methodology and reporting quality of AI prediction models, especially those intended for clinical decision support. One such tool is APPRAISE‐AI, a structured quantitative method to compare the quality of AI studies in the biomedical domain [23]. Other tools include TRIPOD (Transparent reporting of a multivariate prediction model for individual prognosis or diagnosis), a checklist for reporting studies that develop multivariate prediction models using AI, and STARTD‐AI (Standards for Reporting Diagnostic Accuracy Studies‐ AI extension), a statement on how to report studies that use AI for diagnosis [24, 25]. The application of AI has also been investigated in health technology assessment (HTA), particularly economic evaluations, but challenges remain regarding transparency, bias, and accountability [26]. Thus, while AI can enhance data collection and decision‐making processes, ethical concerns must be addressed to ensure responsible implementation of this technology. The limited number of studies on applying AI to measure the quality of health information is a critical gap that needs to be addressed.

Leveraging generative AI's capabilities to calculate quality scores for health‐related social media content seems like an innovative solution to distinguish true from false information. It carries tremendous significance given the rapidly growing body of content on various social media platforms. We sought to address the critical gap in knowledge about how well AI performs compared to humans in rating the quality of health‐related information on social media by investigating Tik Tok videos on IBS. We chose this topic because a previous study from our group had reported on DISCERN scores assessed by two Internal Medicine trained physicians for the 100 most popular Tik Tok videos on this disease [27].

## 2 Methods

The senior author (SC) had previously performed an analysis of the 100 most popular publicly available TikTok videos on IBS and calculated their DISCERN scores [27]. We selected those videos for analysis by AI. We did not obtain any formal ethics approval for this study as all videos were publicly posted, and our research did not involve direct interaction with any content creators or collection of private data. Furthermore, an ethics committee had previously granted an exemption for the prior study using the same dataset, confirming that the research did not meet the criteria for requiring formal ethics approval. The study adhered to ethical guidelines for research involving publicly available content, ensuring that no private or sensitive information was extracted or analyzed beyond what was already accessible to the public.

The sample size for this study was determined based on a previously conducted analysis of the 100 most popular TikTok videos on the topic of IBS, which were scored by two internal medicine physicians specializing in gastroenterology [27]. The study categorized the videos into two groups: those created by individuals with a medical background and those created by individuals without one. Videos created by people with medical backgrounds were given higher DISCERN scores by the physician reviewers. We used this categorization of medical vs. non‐medical background of content creators to compare DISCERN scores given by the two AI models.

The DISCERN instrument comprises of 15 questions (Appendix S1). Each question is scored on a 5‐point scale. A higher score on a question indicates better quality. The average DISCERN score is what we used for our analysis, and we refer to it as the DISCERN score in the paper. Based on the average DISCERN score, we categorized the quality of the audio transcripts as low (1.0–2.5), medium (2.6–3.9), or high quality (4.0–5.0) as described previously [28, 29].

The first step in our analysis was to check each video to see if it was still publicly available. Next, we checked to see if the video contained transcribable spoken audio for the video content. Videos which either featured songs or music that were not suitable for transcription were omitted. This was critical because our analysis required the use of audio transcripts. Once we identified videos with transcribable spoken audio, we individually generated audio transcripts for each of the TikTok videos using the Clap Tools Transcript Generator. We listened to a random sample of videos to verify the accuracy of the audio transcription tool. Once the transcripts for all the videos were generated, an identical prompt was prefixed to each of the transcripts to create a query that was then submitted to the AI models (see Appendix S2 for the audio transcripts for all videos analyzed in this study). Of the three AI models we tested, namely Microsoft Copilot, ChatGPT‐4, and Gemini, only the first two could calculate the DISCERN score and hence were used for this study. By using an identical prompt to prefix each of the transcripts, we ensured consistency of the output of AI DISCERN scoring between the 2 generative AI models.

ChatGPT and Copilot were both used in their default configurations, with no custom settings applied beyond being logged into the respective platforms. OpenAI does not publicly disclose the exact temperature setting used in the ChatGPT web interface; however, based on prior developer documentation and observed model behavior, it is generally understood to operate at a default temperature of approximately 0.7. This setting balances fluency and coherence with moderate variability in responses. Microsoft Copilot, in contrast, does not expose a fixed temperature setting to users and instead dynamically adapts its response style based on user input and context.

We did not implement a context reset between prompts; instead, all interactions were conducted within a continuous session, allowing the model to retain and build upon prior exchanges for both AI models. ChatGPT would not allow us to process the entire 69 videos without being logged in, but CoPilot did not have that constraint. We then ran queries for one TikTok video at a time in each of the AI models and recorded the AI‐generated DISCERN scores in an Excel file (Appendix S2). We calculated the average human DISCERN scores from the individual scores given by the two physician reviewers from the reference article and added that to the same Excel file. The data was imported into and analyzed using SPSS statistical software version 29.0 (IBM Corporation, NY, USA). We compared median DISCERN scores between the AI models and physician reviewers using the Mann–Whitney *U* test. To understand the similarity between human and AI DISCERN scores, we calculated Pearson's correlation coefficient. Using linear regression, we calculated the *R* ^2^ value, a measure of how much an independent variable (AI generated DISCERN score) predicts the dependent variable (physician assigned DISCERN score). A value closer to 1.0 indicates that the independent variable predicts the dependent variable with high certainty. Results were expressed with 95% confidence intervals (CI) where applicable. *p*‐values were two‐sided.

## 3 Results

**FIGURE 1:**
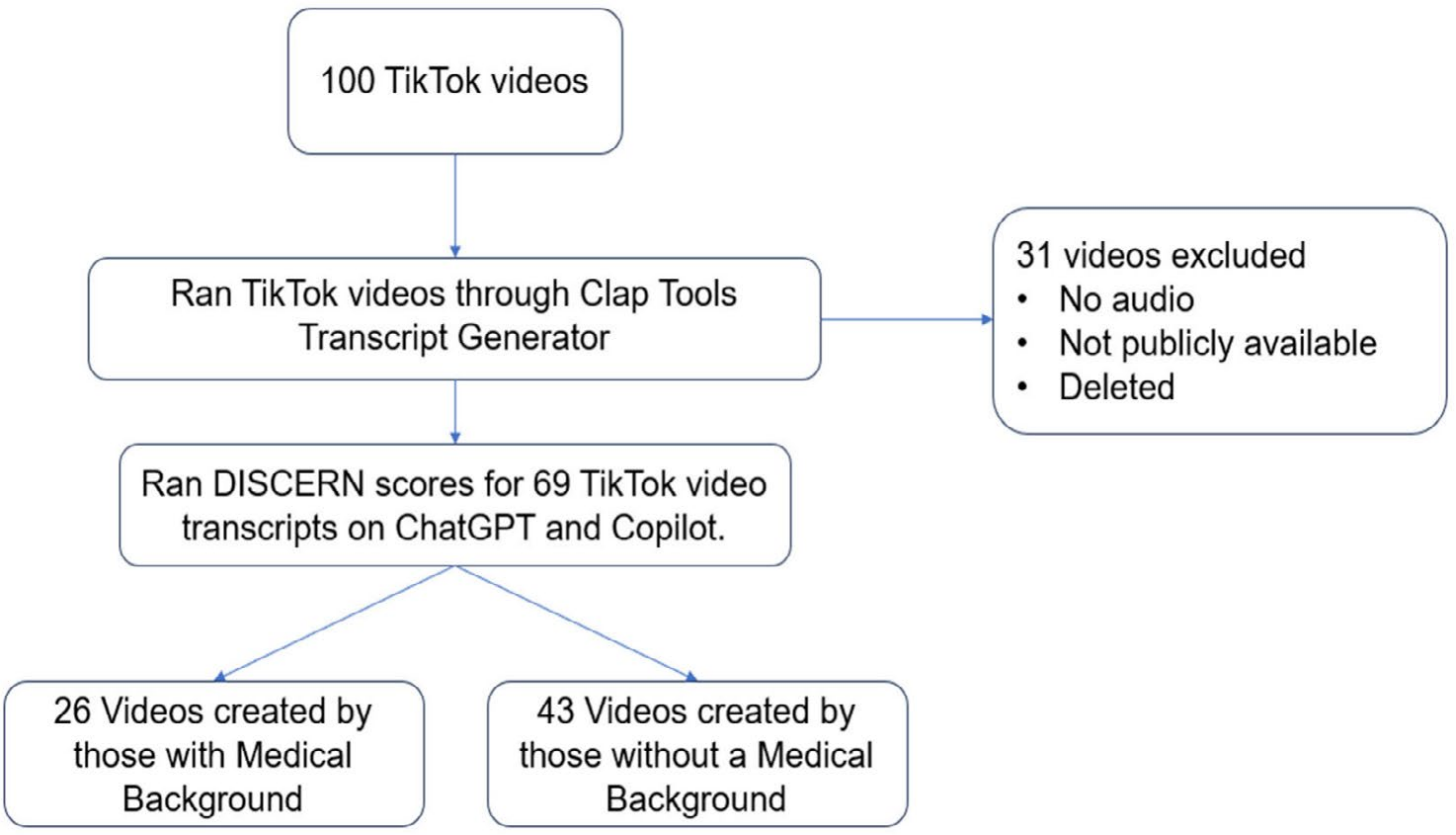
Schematic of the steps in the analysis of TikTok videos.

**FIGURE 2:**
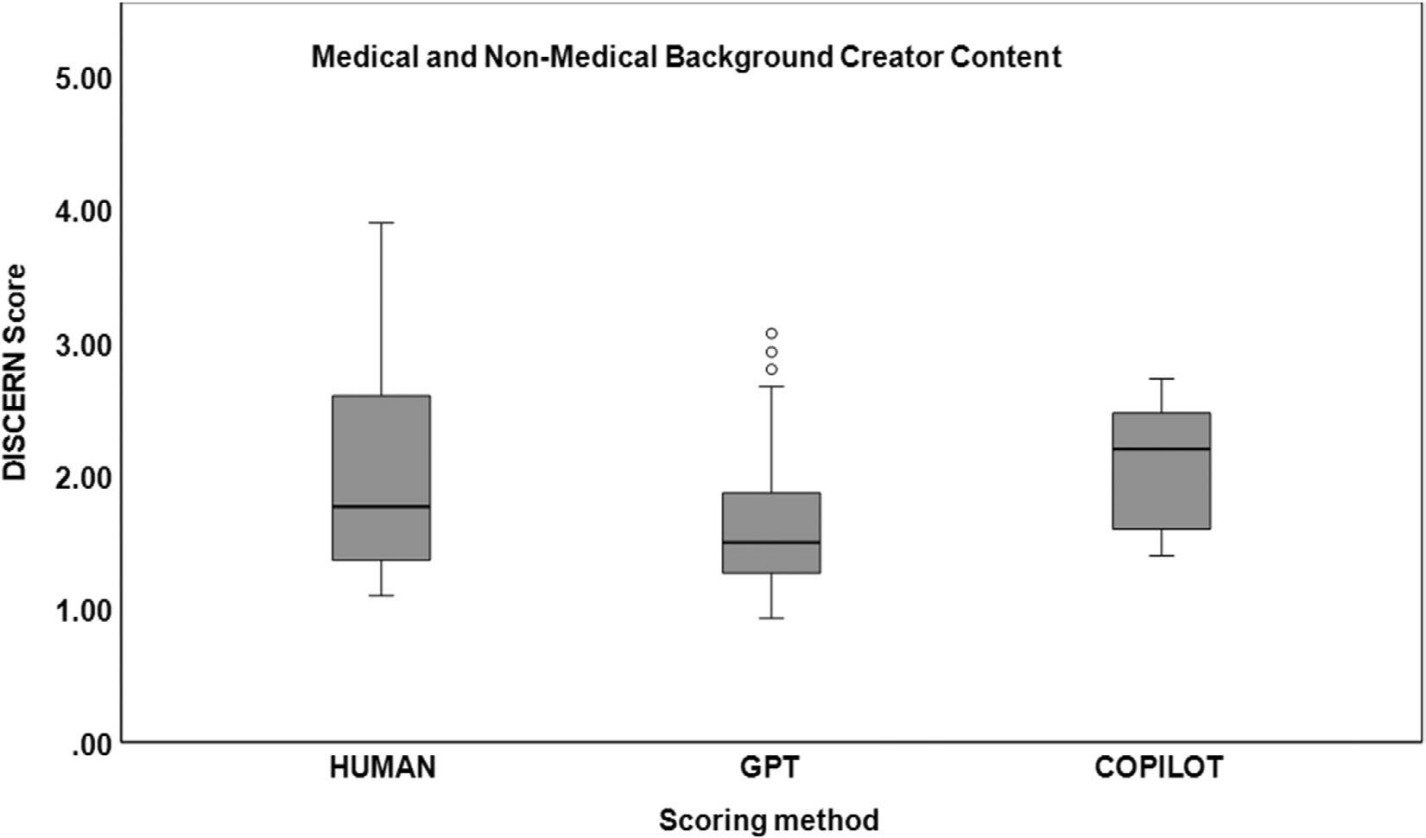
Median DISCERN scores for Human, ChatGPT, and Copilot for all IBS TikTok videos.

Out of 100 videos, 31 were excluded either because of the public non‐availability of the videos or lack of spoken transcribable audio (Figure 1). For the remaining 69 videos, we calculated the DISCERN score. The median DISCERN score for Copilot was significantly higher than that for ChatGPT and humans (Table 1), while the median human DISCERN scores were higher than that for ChatGPT (Figure 2).

**TABLE 1:** Comparison of median DISCERN scores for IBS TikTok videos by Human and AI.

	Median (IQR)	Mann–Whitney *U* test	
		DISCERN GPT	DISCERN Copilot
		*p* value	*p* value
All videos			
DISCERN Human	1.80 (1.25)	< 0.001	0.03
DISCERN GPT	1.50 (0.67)		
DISCERN Copilot	2.20 (0.87)	< 0.001	
Videos by medical creators			
DISCERN Human	2.60 (1.10)	< 0.001	0.47
DISCERN GPT	1.75 (0.53)		
DISCERN Copilot	2.40 (0.25)	< 0.001	
Videos by non‐medical creators			
DISCERN Human	1.50 (0.90)	0.12	< 0.001
DISCERN GPT	1.40 (0.80)		
DISCERN Copilot	2.00 (0.67)	< 0.001	

Videos created by individuals with a medical background received significantly higher DISCERN scores compared to those without a medical background by physician reviewers (*p* < 0.001), ChatGPT (*p* = 0.008) and Copilot (*p* < 0.001). Further, DISCERN scores assigned by human reviewers and Copilot were similar (*p* = 0.47), and both were significantly higher than those assigned by ChatGPT for this group (*p* < 0.001). In contrast, for videos created by non‐medical creators, human and ChatGPT scores did not differ significantly (*p* = 0.12), and both were significantly lower than scores given by Copilot (*p* < 0.001, Table 1).

**FIGURE 3:**
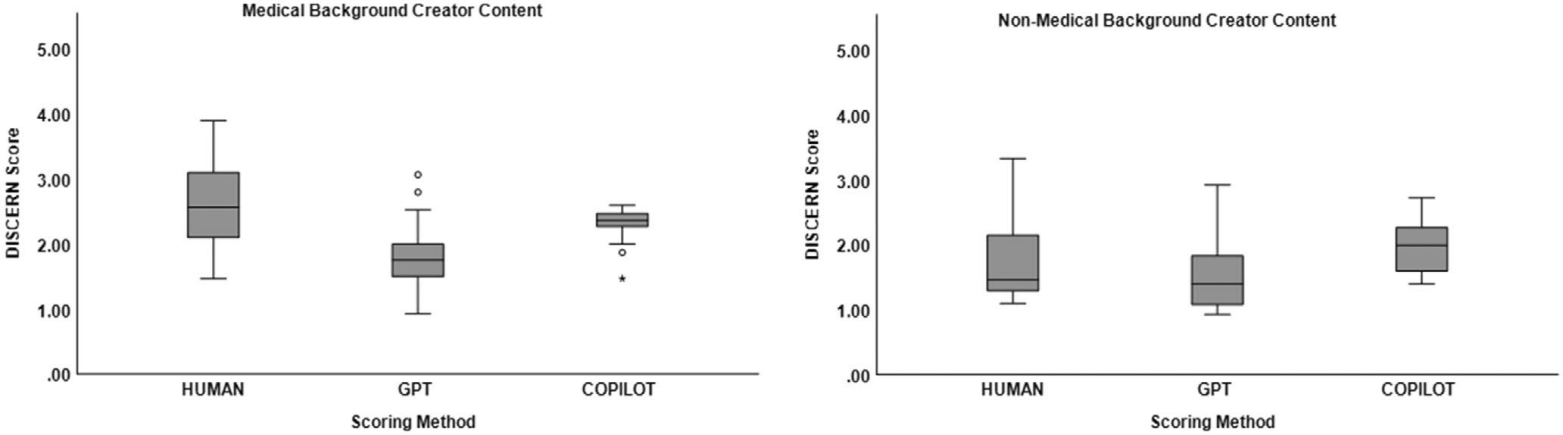
Median DISCERN scores for human, ChatGPT, and Copilot for non‐medical and medical background creator videos, respectively.

Next, we examined whether the DISCERN scores between human, ChatGPT, and Copilot were correlated. There was a strong inter‐rater reliability between the two physician reviewers (*r* = 0.96, 95% CI 0.94–0.98, *p* < 0.001). Overall, AI DISCERN scores were modestly correlated with human DISCERN scores. However, when subdivided into videos by medical and non‐medical creators, there was a stronger correlation for videos made by non‐medical creators (*r* = 0.69–0.75, *p* < 0.001) than for videos by medical creators (i.e., *r* = 0.30–0.31) (Table 2 and Figure 3).

**TABLE 2:** Correlation between Human, ChatGPT, and Copilot.

Dependent variable	Number of videos	Independent variable	Pearson correlation (95% CI)	*p* value
All videos	69			
DISCERN Human		DISCERN ChatGPT	0.60 (0.42–0.73)	< 0.001
DISCERN Human		DISCERN COPILOT	0.65 (0.48–0.77)	< 0.001
DISCERN ChatGPT		DISCERN COPILOT	0.63 (0.46–0.75)	< 0.001
Videos by medical creators	26			
DISCERN Human		DISCERN ChatGPT	0.30 (−0.10–0.61)	0.14
DISCERN Human		DISCERN COPILOT	0.31 (−0.09–0.62)	0.13
DISCERN ChatGPT		DISCERN COPILOT	0.43 (0.05–0.70)	0.03
Videos by non‐medical creators	43			
DISCERN Human		DISCERN ChatGPT	0.75 (0.57–0.86)	< 0.001
DISCERN Human		DISCERN COPILOT	0.69 (0.49–0.82)	< 0.001
DISCERN ChatGPT		DISCERN COPILOT	0.66 (0.45–0.80)	< 0.001

**FIGURE 4:**
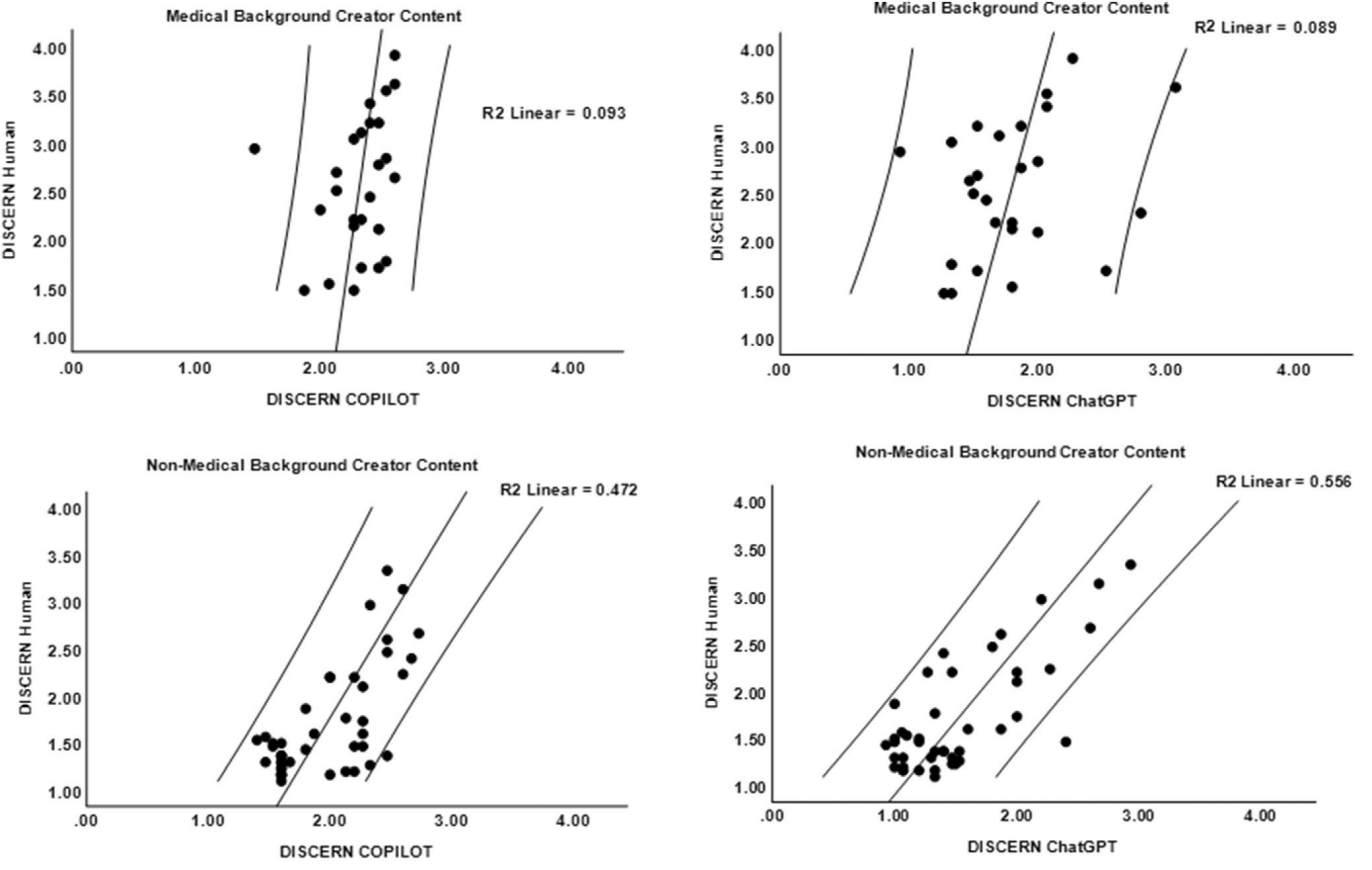
Scatter plot between Human–ChatGPT and Human–Copilot DISCERN scores.

ChatGPT and Copilot DISCERN scores were positively correlated for both categories of videos, with the correlation stronger for non‐medical creators (*r* = 0.66 vs. 0.43, Table 2). Linear regression revealed that AI DISCERN scores predicted between 47% and 56% of the variation in human DISCERN scores for videos created by non‐medical creators. In contrast, the AI DISCERN score did not predict human DISCERN scores for videos by medical content creators (*R* ^2^ < 0.1, Figure 4).

## 4 Discussion

We compared the DISCERN scores for Tik Tok videos on IBS between those assigned by human reviewers and those assigned by ChatGPT and Copilot. Our main finding was that while there was a positive correlation between DISCERN scores given by humans and AI, this was limited to videos created by those without a medical background. Secondly, scores given by the two AI models were also positively correlated but only for videos created by people without a medical background.

Our observation of the difference between human and AI agreement for medical vs. non‐medical content creators supports prior research that AI models can approximate human judgment but struggle with domain‐specific expertise [30]. Studies have found that AI models often misinterpret complex medical terminology. This could impact the scores they assign to health information using DISCERN [31]. These models are also optimized for readability and consumer‐friendly language, making them more aligned with non‐medical creators who use simpler explanations [32]. Medical professionals generally use specialized terminology and complex explanations that AI models may struggle to interpret. Non‐medical creators, on the other hand, tend to structure their content in a way that prioritizes engagement by telling a story, using visual aids, and giving simple explanations, which could lead to higher scores from AI models. This may also lead to bias from AI models towards content that resembles consumer health explanations [33]. AI models rely on pattern recognition and readability metrics and thus may find it easier to assess such non‐technical content compared to highly technical medical videos [34]. They may also lack the nuanced understanding required to assess the credibility of medical information. For example, a meta‐analysis comparing the diagnostic performance of generative AI models and physicians found that these models have a pooled accuracy of approximately 60% (95% CI 51.0%–62.7%). While GPT was better than non‐experts, it performed worse than domain‐specific experts. One hypothesis is that large language AI models (LLMs) currently lack the “common sense” or “intuition” that human physicians develop over years of clinical experience [35]. This inherent weakness of AI may be another reason why these models assign a lower score to videos by medical professionals.

Our findings highlight the limitations of AI models in evaluating the quality of health content. DISCERN score calculation required us to transcribe the audio and present it as a prompt to AI. Several aspects of the videos, such as demographics and credentials of the presenter, voice quality, pitch and modulation, use of visual aids, and body language were not factored into the analysis. In contrast, when physicians evaluated these videos, they were able to factor these into the analysis, or it may have contributed to some bias in their scoring of the videos. Further, AI models often misinterpret complex medical terminology [36]. This stems from AI's reliance on pattern recognition rather than deep contextual understanding. Thus, it may not be suitable for accurately evaluating the quality of highly specialized medical information unless it is trained on those or these are first converted into simpler, non‐technical language before being presented to AI.

The differences in DISCERN scores assigned by ChatGPT and Copilot also highlight the variability in how different AI models interpret the same data. Research has shown that AI models differ in algorithmic design and training methodologies [23]. This variation between models is why we need the standardization of AI‐driven health content assessment improved models trained on domain‐specific medical information. Until we achieve this, human oversight of AI evaluation of medical content is suggested to mitigate the potential risk of misinformation [37].

Our methodology for generating and analyzing transcripts highlights several important considerations regarding AI‐based multimedia analysis. Chief among them is the accuracy of AI‐generated transcripts. The use of Clap Tools Transcript Generator introduces potential accuracy limitations, as AI‐powered speech recognition systems can misinterpret spoken words, particularly in the context of specialized medical language. Prior studies have demonstrated that AI transcription tools have difficulty capturing medical terminology, conversational nuances, and speaker intent accurately [38]. Errors in transcription can affect downstream AI evaluations, leading to discrepancies in DISCERN scores, especially for complex medical discussions. A second issue is the potential bias in how AI processes health information. AI models we used for DISCERN scoring are trained on publicly available data, which may introduce biases due to these models being optimized for improving readability, leading to more consistent scores for non‐technical medical content [9]. There can also be variability in how AI models interpret the same transcript. There are also ethical and practical concerns when using AI to assess health content, particularly the chances of AI making incorrect conclusions based on health information. Current research supports hybrid AI‐human evaluation strategies to mitigate bias and improve accuracy while assessing online health content [39].

ChatGPT assigned DISCERN scores accounted for a greater proportion of variance in human DISCERN scores (55.6%) than Copilot (47.2%) for videos by non‐medical creators. This difference may reflect how different AI models processed the transcripts. ChatGPT is designed for general, consumer‐friendly language, making it more aligned with non‐medical creators [40]. Copilot, on the other hand, is designed to process technical and structured information, making it more likely to be aligned to medically complex content [41]. Additionally, the two AI models may interpret DISCERN scores differently. While DISCERN scores assess the quality and reliability of health information, AI models may emphasize distinct aspects in their evaluations—ChatGPT tends to prioritize readability and accessibility, whereas Copilot focuses more on depth and evidence‐based accuracy, leading to assessments that resemble expert evaluations. This distinction suggests that ChatGPT may be more suitable for evaluating health information written by individuals without a medical background, while Copilot may be better suited for assessing content created by medical professionals [42]. Future research is needed to validate this hypothesis and explore the implications of AI‐driven health information assessments.

AI‐generated DISCERN scores have several promising applications in health information assessment. First, social media platforms could integrate these scores to help users evaluate the credibility of health‐related content, ensuring greater awareness of its quality. Second, health content creators can leverage AI DISCERN evaluations to refine their material, enhancing clarity and readability based on AI‐driven feedback. AI DISCERN scores could identify misleading health information, improving patient education materials and optimizing content for accuracy and readability [43].

Despite its novelty, our study has several limitations. The sample size was relatively small, and the videos analyzed focused on a single disease state, presented in a single language (English), and hosted on a single platform (TikTok). While DISCERN is a validated tool for assessing written health information, its application to AI‐generated transcripts of video content requires further investigation. TikTok videos are typically brief—often under 1 min—limiting the depth of health information conveyed. How this approach performs for longer videos, for instance on YouTube, remains to be seen. Relying on English‐language transcripts may introduce bias, as TikTok is a global platform with creators producing content in multiple languages. Applying this methodology to translated transcripts from other languages may allow us to gain a broader understanding of AI's role in the assessment of health information quality. AI systems, including large language models like GPT and Copilot, encounter significant obstacles in real‐world applications due to phenomena such as hallucinations and overestimation bias. Hallucinations occur when AI models generate seemingly plausible yet factually incorrect or entirely fabricated information. Overestimation bias occurs when AI models exhibit unreasonable confidence in their responses, even when incorrect. To mitigate this problem, several strategies have been suggested, such as automated reasoning to improve logical consistency, retrieval‐augmented generation to ground responses in reliable sources, and bias auditing to improve transparency and fairness [44, 45].

An extension of our study could be to use the DISCERN score to evaluate the quality of AI‐generated education materials, identify misinformation, and better analyze patient education materials for accuracy and readability. For instance, a recent study comparing responses from ChatGPT and Copilot to questions about guidelines on the management of pancreatic cancer found that ChatGPT was more accurate than Copilot (52% vs. 33%) [46].

In summary, our research contributes to the ongoing discussion on AI's role in health content evaluation, highlighting both its potential and limitations. While AI models demonstrate moderate correlation with human assessments, their effectiveness varies based on content complexity and creator background. Future research should explore AI's performance across diverse health topics, languages, and social media platforms to refine its evaluation capabilities and improve content reliability. Refining AI training datasets, improving transcript processing capabilities, and minimizing biases in AI‐driven scoring systems can improve confidence in the reliability of AI‐assisted health content assessments.

## Author Contributions

Dhruva Arcot contributed to the conceptualization of the study, data collection, and initial drafting of the manuscript. Neha Pondicherry contributed to conceptualization of the study, data collection, data analysis, interpretation of results, and critical revision of the manuscript for important intellectual content. Subhankar Chakraborty supervised the project, contributed to the study design and methodology, assisted with data analysis and interpretation of the results, and provided substantial input in manuscript editing and final approval of the version to be published. All authors reviewed and approved the final manuscript.

## Disclosure

The authors have nothing to report.

## Conflicts of Interest

The authors declare no conflicts of interest.

## Supporting information


**Appendix S1:** DISCERN test questions.

**Appendix S2:** Comparison of DISCERN scores for TikTok videos on irritable bowel syndrome (IBS), as rated by two independent physician reviewers, ChatGPT, and Microsoft Copilot. Columns include reviewer background, individual and average DISCERN scores, and transcripts evaluated. DISCERN scores are reported on a 5‐point scale (higher scores = higher quality).

## Data Availability

The data that supports the findings of this study are available in the Supporting Information of this article.

## References

[1] Statistics NCfH , “Health Information on the Internet: Centers for Disease Control and Prevention,” (2022).

[2] Maine Uo , “Social Media Statistics and Details: Undiscovered Maine,” (2024).

[3] Health HP , Social Media and Health: From Misinformation to Education (Harvard Public Health, 2024).

[4] M. Gruber , “Social Media and Health Misinformation: A Literature Review,” in *Proceedings of the Future Technologies Conference (FTC)*, volume 3, ed. K. Arai (Springer, 2024), 345–359.

[5] B. D. Menz , N. D. Modi , M. J. Sorich , and A. M. Hopkins , “Health Disinformation Use Case Highlighting the Urgent Need for Artificial Intelligence Vigilance: Weapons of Mass Disinformation,” JAMA Internal Medicine 184 (2024): 92–96. 37955873 10.1001/jamainternmed.2023.5947

[6] B. D. Menz , N. M. Kuderer , S. Bacchi , et al., “Current Safeguards, Risk Mitigation, and Transparency Measures of Large Language Models Against the Generation of Health Disinformation: Repeated Cross Sectional Analysis,” BMJ 384 (2024): e078538. 38508682 10.1136/bmj-2023-078538 PMC10961718

[7] H. R. Saeidnia , E. Hosseini , B. Lund , M. A. Tehrani , S. Zaker , and S. Molaei , “Artificial Intelligence in the Battle Against Disinformation and Misinformation: A Systematic Review of Challenges and Approaches,” Knowledge and Information Systems 67 (2025): 3139–3158.

[8] I. Rowlands , D. Loxton , A. Dobson , I. J. Rowlands , and G. D. Mishra , “Seeking Health Information Online: Association With Young Australian Women's Physical, Mental, and Reproductive Health,” Journal of Medical Internet Research 17 (2015): e120. 25986630 10.2196/jmir.4048 PMC4468597

[9] M. Ghassemi and A. Gusev , “Limiting Bias in AI Models for Improved and Equitable Cancer Care,” Nature Reviews Cancer 24 (2024): 823–824. 10.1038/s41568-024-00739-x 39191902

[10] American Public Health A , “Criteria for Assessing the Quality of Health Information on the Internet,” American Journal of Public Health 91 (2001): 513–514. 11236453 10.2105/ajph.91.3.513 PMC1446565

[11] D. Charnock , S. Shepperd , G. Needham , and R. Gann , “DISCERN: An Instrument for Judging the Quality of Written Consumer Health Information on Treatment Choices,” Journal of Epidemiology and Community Health 53 (1999): 105–111. 10396471 10.1136/jech.53.2.105 PMC1756830

[12] Applied Health Research Unit UoO , “DISCERN: A Tool for Assessing Health Information,” (2025).

[13] P. L. Gorrepati and G. P. Smith , “Evaluating Social Media as a Source of Patient Information Regarding Psoriasis Treatments Using the DISCERN Instrument,” Journal of Dermatological Treatment 33 (2022): 2685–2686. 35107049 10.1080/09546634.2022.2037497

[14] R. D'Ambrosi , A. Annibaldi , A. Carrozzo , et al., “Evaluating the Reliability of YouTube as a Source of Information for Meniscal Ramp Lesions,” Orthopaedic Journal of Sports Medicine 12, no. 1 (2024): 23259671231219815. 38188623 10.1177/23259671231219815 PMC10768595

[15] T. Subramanian , K. Araghi , I. Akosman , et al., “Quality of Spine Surgery Information on Social Media: A DISCERN Analysis of TikTok Videos,” Neurospine 20 (2023): 1443–1449. 38171310 10.14245/ns.2346700.350 PMC10762400

[16] A. C. Fullard , S. M. Johnston , and D. J. Hehir , “Quality and Reliability Evaluation of Current Internet Information Regarding Mesh Use in Inguinal Hernia Surgery Using HONcode and the DISCERN Instrument,” Hernia 25 (2021): 1325–1330. 33852079 10.1007/s10029-021-02406-8

[17] B. D. Rust , J. Choudhari , E. Christoforides , et al., Quality Analysis of Patient Educational TikTok Videos for Knee Instability (American Academy of Physical Medicine and Rehabilitation, 2023).

[18] S. Ming , J. Han , M. Li , Y. Liu , K. Xie , and B. Lei , “TikTok and Adolescent Vision Health: Content and Information Quality Assessment of the Top Short Videos Related to Myopia,” Frontiers in Public Health 10 (2022): 1068582. 36684892 10.3389/fpubh.2022.1068582 PMC9845771

[19] M. Arslan , C. Cottone , E. Mangona , A. Rafizadeh , M. Mohsin , and J. Frey , “Microtia and Social Media: How Can we Help Our Patients?,” Journal of Craniofacial Surgery 35 (2024): 2113–2115. 39226410 10.1097/SCS.0000000000010590

[20] IBM , Artificial Intelligence (IBM, 2025).

[21] IBM , “Generative AI,” (2025).

[22] X. Liu , L. He , E. Alanazi , E. Liu , A. Goss , and L. Gumireddy , “Assessing the Accuracy and Explainability of Using ChatGPT to Evaluate the Quality of Health News,” BMC Public Health 25 (2025): 2038. 40457340 10.1186/s12889-025-23206-0 PMC12128262

[23] J. C. C. Kwong , A. Khondker , K. Lajkosz , et al., “APPRAISE‐AI Tool for Quantitative Evaluation of AI Studies for Clinical Decision Support,” JAMA Network Open 6 (2023): e2335377. 37747733 10.1001/jamanetworkopen.2023.35377 PMC10520738

[24] K. G. Moons , D. G. Altman , J. B. Reitsma , et al., “Transparent Reporting of a Multivariable Prediction Model for Individual Prognosis or Diagnosis (TRIPOD): Explanation and Elaboration,” Annals of Internal Medicine 162 (2015): W1–W73. 25560730 10.7326/M14-0698

[25] P. M. Bossuyt , J. B. Reitsma , D. E. Bruns , et al., “STARD 2015: An Updated List of Essential Items for Reporting Diagnostic Accuracy Studies,” BMJ 351 (2015): h5527. 26511519 10.1136/bmj.h5527 PMC4623764

[26] M. Ramezani , A. Bakhtiari , R. Daroudi , et al., “Applications of Artificial Intelligence and the Challenges in Health Technology Assessment: A Scoping Review and Framework With a Focus on Economic Dimensions,” Health Economics Review 15 (2025): 46. 40461901 10.1186/s13561-025-00645-4 PMC12135449

[27] G. Waidyaratne , J. Daboul , S. Liyanarachchi , and S. Chakraborty , “The Evaluation and Analysis of Irritable Bowel Syndrome‐Related Short Videos on Social Media (TikTok),” Alimentary Pharmacology and Therapeutics 60 (2024): 350–356. 38853598 10.1111/apt.18096

[28] D. Charnock , S. Shepperd , G. Needham , and R. Gann , “An Instrument for Judging the Quality of Written Consumer Health Information on Treatment Choices,” Journal of Epidemiology and Community Health 53, no. 2 (1999): 105–111. https://doi.org/10.1136/jech.53.2.105. 10396471 10.1136/jech.53.2.105 PMC1756830

[29] D. Charnock , S. Shepperd , G. Needham , and R. Gann , Discern Handbook (University of Oxford, 1999). 10.1136/jech.53.2.105 PMC1756830 10396471

[30] J. Yun , “Meta‐Analysis of Inter‐Rater Agreement and Discrepancy Between Human and Automated English Essay Scoring,” English Teaching 78 (2023): 105–124.

[31] Y. K. Ghanem , A. D. Rouhi , A. Al‐Houssan , et al., “Dr. Google to Dr. ChatGPT: Assessing the Content and Quality of Artificial Intelligence‐Generated Medical Information on Appendicitis,” Surgical Endoscopy 38 (2024): 2887–2893. 38443499 10.1007/s00464-024-10739-5 PMC11078845

[32] Y. Semerci , “Evaluation of the Reliability and Readability of Chatgpt‐4.0 Responses Regarding Complaint of Dizziness Cases,” Scholars Journal of Applied Medical Sciences 13 (2025): 83–87.

[33] Y. Tao , O. Viberg , R. S. Baker , and R. F. Kizilcec , “Cultural Bias and Cultural Alignment of Large Language Models,” PNAS Nexus 3 (2024): pgae346. 39290441 10.1093/pnasnexus/pgae346 PMC11407280

[34] F. G. Rebitschek , A. Carella , S. Kohlrausch‐Pazin , M. Zitzmann , A. Steckelberg , and C. Wilhelm , “Evaluating Evidence‐Based Health Information From Generative AI Using a Cross‐Sectional Study With Laypeople Seeking Screening Information,” npj Digital Medicine 8 (2025): 343. 40490558 10.1038/s41746-025-01752-6 PMC12149300

[35] H. Takita , D. Kabata , S. L. Walston , et al., “A Systematic Review and Meta‐Analysis of Diagnostic Performance Comparison Between Generative AI and Physicians,” npj Digital Medicine 8, no. 1 (2025): 175. 10.1038/s41746-025-01543-z. 40121370 PMC11929846

[36] C. J. Kelly , A. Karthikesalingam , M. Suleyman , G. Corrado , and D. King , “Key Challenges for Delivering Clinical Impact With Artificial Intelligence,” BMC Medicine 17 (2019): 195. 31665002 10.1186/s12916-019-1426-2 PMC6821018

[37] National Institutes of H , “NIH Findings Shed Light on Risks and Benefits of Integrating AI Into Medical Decision‐Making,” (2024).

[38] M. Ibrahim , Y. A. Khalil , S. Amirrajab , et al., “Generative AI for Synthetic Data Across Multiple Medical Modalities: A Systematic Review of Recent Developments and Challenges,” Computers in Biology and Medicine 189 (2025): 109834. 40023073 10.1016/j.compbiomed.2025.109834

[39] A. Chen , L. Liu , and T. Zhu , “Advancing the Democratization of Generative Artificial Intelligence in Healthcare: A Narrative Review,” Journal of Hospital Management and Health Policy 8 (2024): 8.

[40] A. R. Swisher , A. W. Wu , G. C. Liu , M. K. Lee , T. R. Carle , and D. M. Tang , “Enhancing Health Literacy: Evaluating the Readability of Patient Handouts Revised by Chatgpt's Large Language Model,” Otolaryngology and Head and Neck Surgery 171 (2024): 1751–1757. 10.1002/ohn.927 39105460

[41] G. Rossettini , L. Rodeghiero , F. Corradi , et al., “Comparative Accuracy of ChatGPT‐4, Microsoft Copilot and Google Gemini in the Italian Entrance Test for Healthcare Sciences Degrees: A Cross‐Sectional Study,” BMC Medical Education 24 (2024): 694. 38926809 10.1186/s12909-024-05630-9 PMC11210096

[42] M. Yoo and C. W. Jang , “Presentation Suitability and Readability of Chatgpt's Medical Responses to Patient Questions About on Knee Osteoarthritis,” Health Informatics Journal 31, no. 1 (2025): 14604582251315587. 39828887 10.1177/14604582251315587

[43] D. Armstrong , C. Paul , B. McGlaughlin , and D. Hill , “Can Artificial Intelligence (AI) Educate Your Patient? A Study to Assess Overall Readability and Pharmacists' Perception of AI‐Generated Patient Education Materials,” JACCP: Journal of the American College of Clinical Pharmacy 7 (2024): 803–808.

[44] F. Hasanzadeh , C. B. Josephson , G. Waters , D. Adedinsewo , Z. Azizi , and J. A. White , “Bias Recognition and Mitigation Strategies in Artificial Intelligence Healthcare Applications,” npj Digital Medicine 8 (2025): 154. 40069303 10.1038/s41746-025-01503-7 PMC11897215

[45] Y. Sun , D. Sheng , Z. Zhou , and Y. Wu , “AI Hallucination: Towards a Comprehensive Classification of Distorted Information in Artificial Intelligence‐Generated Content,” Humanities and Social Sciences Communications 11 (2024): 1278.

[46] K. N. Kaiser , A. J. Hughes , A. D. Yang , et al., “Use of Large Language Models as Clinical Decision Support Tools for Management Pancreatic Adenocarcinoma Using National Comprehensive Cancer Network Guidelines,” Surgery 182 (2025): 109267. 40055080 10.1016/j.surg.2025.109267 PMC13184376

