# Supplementary material for: Evaluating the Quality of Health Information: Comparison of Human and Artificial Intelligence
Source: Neurogastroenterol Motil. 2025 Sep 24;38(6):e70164. doi: 10.1111/nmo.70164 (PMC13244121; doi:10.1111/nmo.70164)
Supplement: Supplementary file 1 — Appendix S1: DISCERN test questions. [file NMO-38-e70164-s001.docx]

**Appendix 1 DISCERN test questions.**

| **Question Number** | **Question** |
| --- | --- |
| 1 | Are the aims clear? |
| 2 | Does it achieve its aims? |
| 3 | Is it relevant? |
| 4 | Is it clear what sources of information were used to compile the publication (other than the author or producer)? |
| 5 | Is it clear when the information used or reported in the publication was produced? |
| 6 | Is it balanced and unbiased? |
| 7 | Does it provide details of additional sources of support and information? |
| 8 | Does it refer to areas of uncertainty? |
| 9 | Does it describe how each treatment works? |
| 10 | Does it describe the benefits of each treatment? ￼ |
| 11 | Does it describe the risks of each treatment? |
| 12 | Does it describe what would happen if no treatment is used? |
| 13 | Does it describe how treatment choices affect overall quality of life? |
| 14 | Is it clear that there may be more than one possible treatment option? |
| 15 | Does it provide support for shared decision-making? |
